# Blind testing in firearms: Preliminary results from a blind quality control program

**DOI:** 10.1111/1556-4029.15031

**Published:** 2022-03-29

**Authors:** Maddisen Neuman, Callan Hundl, Aimee Grimaldi, Donna Eudaley, Darrell Stein, Peter Stout

**Affiliations:** ^1^ Houston Forensic Science Center Houston Texas USA; ^2^ Center for Statistics and Applications in Forensic Evidence (CSAFE) Ames Iowa USA

**Keywords:** blind quality control, blind testing, firearms comparison conclusions, firearms examination, inconclusive rate, proficiency testing, quality improvement

## Abstract

Open proficiency tests meet accreditation requirements and measure examiner competence but may not represent actual casework. In December 2015, the Houston Forensic Science Center began a blind quality control program in firearms examination. Mock cases are created to mimic routine casework so that examiners are unaware they are being tested. Once the blind case is assigned to an examiner, the evidence undergoes microscopic examination and comparison to determine whether the fired evidence submitted was fired in the same firearm. Fifty‐one firearms blind cases resulting in 570 analysis and comparison determinations were reported between December 2015 and June 2021. No unsatisfactory results were obtained; however, 40.3% of comparisons in which the ground truth was either elimination or identification resulted in inconclusive conclusions. Due to the quality of some of the evidence submitted, inconclusive results were not unexpected. A ground truth of elimination and comparison result of inconclusive was observed at a rate of 74%, while a ground truth of identification and comparison result of inconclusive was observed at a rate of 31%. Bullets (61.8%) were the main contributors to inconclusive conclusions; variables such as the assigned examiners, training program, examiner experience, and the intended complexity of the case did not significantly contribute to the results. The program demonstrates that the quality management system and firearms section procedures can obtain accurate and reliable results and provides examiners added confidence in court. Additionally, the program can be tailored to target specific research questions and provide opportunities for collaboration with other laboratories and researchers.

**Highlights**

- Initial findings from a blind testing program in firearms examination are presented.
- No identifications declared for nonmatching pairs; no eliminations declared for matching pairs.
- 40.3% of comparisons (ground truth identification or elimination) were determined to be inconclusive.
- Bullets were the main contributors to inconclusive results (61.8%).
- Benefits and limitations of a blind testing program in firearms examination are discussed.

## 1 INTRODUCTION

Proficiency testing is a requirement for accredited forensic science service providers and serves an important role in the ability to confirm adequate competence among individual analysts and across laboratories. Most proficiency tests are prepared by a vendor, and the results are unknown to the participant but are “open,” meaning the forensic practitioners are aware that they are being tested. Open proficiency tests are tools for assessing the performance of analytical steps and providing a means by which to conduct interlaboratory comparisons. However, proficiency tests do not mimic routine casework of the laboratory in packaging, paperwork, or distribution. Despite these differences, analysts are asked to work these proficiency tests as routine casework, which may inflate accuracy rates [1, 2, 3]. Scholars have noted the lack of difficulty in proficiency tests [4, 5, 6, 7, 8] and found that analysts may behave differently during proficiency testing than during routine casework [9, 10], an example of the phenomenon known as the Hawthorne effect [11].

In 2009, the National Academy of Sciences (NAS) published a report that described the current state of forensic science practice and outlined recommendations for many forensic science disciplines [12]. The report recommended blind proficiency testing as a more precise test of a worker's accuracy. Scholars have reiterated the NAS report's sentiments and called for widespread use of blind proficiency testing [5, 13, 14, 15]. Analysis of proficiency testing has suggested that blind testing can reduce error rates by as much as 46%, depending on the level of bias and potential for penalties received by the test taker [16]. Blind testing also capitalizes on the idea of the Hawthorne effect by providing a scenario in which potential bias associated with proficiency testing is controlled and reduced. Despite continued calls to implement blind testing in forensic science, to the authors' knowledge, few forensic laboratories have implemented blind testing and published the results [17, 18, 19].

The Houston Forensic Science Center (HFSC) is a local government corporation that operates independently from law enforcement. In September 2015, HFSC implemented a blind quality control (blind QC) program. The program was initiated in the toxicology section and has expanded over the years to include the seized drugs, latent prints, forensic biology, multimedia, and firearms sections. The intent of the blind QC program is to supplement open proficiency tests required for accreditation; HFSC is accredited to the International Organization for Standardization/International Electrotechnical Commission (ISO/IEC) 17,025:2017 standard by the American National Standards Institute (ANSI) National Accreditation Board (ANAB). The program is facilitated and maintained by HFSC's quality division, which is organizationally separate from the laboratory sections; as such, blind QC cases are prepared and introduced into the workflow by personnel who are not associated with the testing. Blind QC cases are created to mimic real casework with the intent that the analysts will be unaware that the cases are mock cases and give the cases no special treatment.

Blind testing was introduced in the firearms section in December 2015. Firearms blind QC cases are intended to be submitted and packaged in a similar manner to the casework seen by the firearms section. Blind QC cases are submitted at a rate that equals approximately 5% of the monthly firearms examination case output average from the previous year. The goal was implemented in the Firearms section in mid‐2018, equating to one blind QC submission per month.

This manuscript:

1. Describes preliminary results from a blind testing program in firearms examination.
2. Examines the prevalence of examiner conclusions and explores the extent to which there are trends related to examiners and examiner conclusions.
3. Discusses the benefits the firearms section garners from the blind QC program.

## 2 MATERIALS AND METHODS

### 2.1 Firearms procedures

The firearms section conducts casework on a request basis from stakeholders. The majority of requests are for microscopic examination and comparison of fired bullets and/or cartridge cases to determine whether the fired evidence was fired in the same firearm. Fired evidence also includes shotshells, shot pellets, shot carriers, and fragments. Unfired cartridges, or live rounds, may be submitted as evidence. Since live rounds are not fired in a firearm, they are not typically submitted for comparison purposes; however, unfired cartridges may be used to create test fires when the submitted unfired cartridges match the ammunition type of the submitted fired evidence. The firearms section also operates a program for the National Integrated Ballistic Information Network (NIBIN). This program is not request based; instead, firearms suitable for NIBIN processing are automatically submitted. NIBIN technicians test fire firearms for NIBIN entry. The fired bullets and cartridge cases created from test firing may be used as known samples for examination in casework.

If a firearm is submitted, an examiner fires the firearm to test functionality and create a set of test fires. The test fires, which are a set of cartridge cases and bullets known to have been fired in a firearm (i.e., known samples), can be compared to the fired evidence submitted in the case (i.e., unknown samples). Firearms examiners use comparison microscopes to compare two items (e.g., bullets or cartridge cases) side by side. Items are examined for markings made during the firing process, and conclusions are rendered based on the level of agreement or disagreement of these marks. Additional microscopic examinations on fired evidence include determining what types of firearms may have fired the evidence.

Based on the Association of Firearms and Tool Mark Examiners (AFTE) range of conclusions [20], the firearms section's range of conclusions includes *Identification*, *Elimination*, *Inconclusive*, and *Unsuitable*. Additionally, firearms examiners at HFSC may conclude that items of evidence are *Insufficient* for analysis. The HFSC firearms section interprets unsuitable to mean that an item has no markings created by a firearm. The non‐standard conclusion of insufficient is used to record more information regarding an item and/or provide additional information to the stakeholder. Unsuitable and insufficient conclusions are suitability determinations, whereas identification, elimination, and inconclusive are comparison conclusions. Conclusions of identification are based on individual characteristics, while conclusions of elimination can be based on class or individual characteristics. An inconclusive conclusion indicates an inadequate correspondence of individual and/or class characteristics needed to make an identification or elimination decision. Table 1 provides more detail on the firearms section's range of conclusions as written in the firearms section range of conclusions document [21].

**TABLE 1:** Firearms analysis range of conclusions

Identification^a^	A sufficient correspondence of individual characteristics will lead the examiner to the conclusion that both items (evidence and tests) originated from the same source.
Elimination	A disagreement of class characteristics will lead the examiner to the conclusion that the items did not originate from the same source. In some instances, it may be possible to support a finding of elimination even though the class characteristics are similar when there is marked disagreement of individual characteristics.
Inconclusive	An insufficient correspondence of individual and/or class characteristics will lead the examiner to the conclusion that no identification or elimination could be made with respect to the items examined.
Unsuitable	A lack of suitable microscopic characteristics will lead the examiner to the conclusion that the items are unsuitable for identification.
Insufficient	Examiners may render an opinion that markings on an item are insufficient when: An item has discernible class characteristics but no individual characteristics. An item does not exhibit class characteristics and has few individual characteristics of such poor quality that precludes an examiner from rendering an opinion. The examiner cannot determine if markings on an item were made by a firearm during the firing process. The examiner cannot determine if markings are individual or subclass.

a The identification of cartridge case/bullet toolmarks is made to the practical, not absolute, exclusion of all other firearms. This is because it is not possible to examine all firearms in the world, a prerequisite for absolute certainty. The conclusion that sufficient agreement for identification exists between toolmarks means that the likelihood that another firearm could have made the questioned toolmarks is so remote as to be considered a practical impossibility.

Class characteristics refer to features of firearms that are under the control of the manufacturer of the firearm (e.g., the number and twist of the lands and grooves in a barrel or the shape of the firing pin). Subclass characteristics are features that may be produced during manufacture that are consistent among items fabricated by the same tool in the same approximate state of wear [20]. These features are not determined prior to manufacture and are more restrictive than class characteristics but less restrictive than individual characteristics. Individual characteristics are marks unique to a firearm, which occur beyond the control of manufacture (e.g., ever‐changing tool edges and multiple manufacturing techniques used on the same item). Class and individual characteristics of firearms are imparted onto bullets and cartridge cases when a firearm is fired.

Some case requests, such as firearm functionality testing and single items submitted for rifling characteristic analysis, do not require a second examiner, but are technically and administratively reviewed by two additional firearms examiners. Every case in which comparisons are conducted or in which the item(s) is deemed unsuitable or insufficient for comparison is examined by a secondary examiner in a process called verification. When a case requires a second examiner, the second examiner conducts an administrative and technical review before a third examiner also technically and administratively reviews the case. Should the primary and second examiner reach different conclusions during examination or verification, the examiners would follow the section's consultation and conflict resolution policy, which was put into practice in 2018 [22].

### 2.2 Firearms blind QC procedures

The firearms blind QC cases are designed and submitted in a manner consistent with the evidence items and offense types that the section observes in routine casework. Most of the casework received by HFSC is submitted by the Houston Police Department (HPD), so understanding what HPD submits on a regular basis is integral for creating blind QC cases that most closely mimic HPD submissions in packaging, submission process, and offense type. In routine casework, HPD enters cartridge case evidence into NIBIN prior to the evidence being submitted to HFSC for examination. The blind QC program must bypass this process in order to keep mock evidence from being uploaded to NIBIN. Instead, blind QC evidence is packaged in a way that mimics HPD’s NIBIN procedures without going through this process. See Hundl et al. (2019) [23] for more detail regarding the creation of blind QC cases and the program's overall benefit to HFSC.

Fired evidence is created using firearms slated by HPD for destruction, HFSC staff's personally owned firearms, or firearms from HFSC's reference collection (a library of firearms used for parts and training). The firearm(s) used to create the fired evidence may or may not be submitted as an item of evidence. When more than one firearm is used to create fired evidence, bullets and cartridge cases are marked with an ultraviolet (UV) pen or otherwise made identifiable by documenting unique features. Marking the evidence that was created from one firearm allows the firearms section manager and/or the quality division to review the evidence after analysis and determine whether ground truth was reached in the case. Since determining how the analyst will itemize the evidence is not possible, marking the fired evidence with a UV pen is a way to keep track of which item was fired from a particular firearm. The markings from the UV pen will remain invisible to the examiner through the course of examination. If a piece of fired evidence has distinguishing features that the firearms manager or the quality division can use to identify the evidence after analysis, then marking the evidence with a UV pen may be unnecessary.

Since the blind QC evidence must mimic normal casework to appear authentic to the examiner, a variety of samples are submitted. Not all items submitted are intended to be suitable for comparison, such as bullet fragments, bullet cores, and other items which the examiner may conclude are insufficient or unsuitable for comparison due to quality. Additionally, some items are intentionally submitted to make comparisons challenging. For example, Glocks, which were used to create evidence for four blind QC cases, are known in the firearms community to poorly mark bullets due to the method Glock uses to rifle their barrels (i.e., hammer forging that can result in polygonal rifling). Another way in which the examiners can be challenged is by submitting fired evidence created using more than one firearm with the same class characteristics. Two hundred and ninety (51%) comparisons were created with two different firearms of the same class. These comparisons are challenging because class characteristics will be the same, but individual characteristics will not; thus, the ground truth will be elimination despite class characteristic similarities. Open proficiency test consensus results are typically either identification or elimination conclusions, providing few circumstances in which examiners might determine inconclusive. HFSC can mitigate the lack of inconclusive consensus results in proficiency tests by submitting blind QC items with a range of complexity to further test the firearms workflow.

Firearms section management evaluates the created evidence prior to submission to determine the expected results and reviews the results of the completed blind QC cases to determine satisfactory completion. A satisfactory result may include: (1) a result that conforms to the known ground truth, or (2) a result that does not necessarily conform to the known ground truth but is technically sound (i.e., a known elimination/identification that is reported as inconclusive based on the applicable standards in the field) [24]. A firearms examiner should conclude inconclusive if the item does not contain the quality or quantity of information needed to include or exclude from another item. Lack of individual characteristics could be due to factors such as a firearm or ammunition that does not mark well or damage to the item after firing. Because some poor‐quality items are submitted intentionally to be challenging and elimination on individual characteristics alone is more difficult, inconclusive conclusions are expected. An inconclusive result is an acceptable conclusion based on criteria outlined in the AFTE range of conclusions [20] and the firearms section range of conclusions document [21] and is not considered an error.

Fragments and bullet cores are submitted as ground truths of unsuitable or insufficient regardless of which firearm was used to create the evidence. Because all suitability and comparison conclusions are verified by an additional examiner, all conclusions are the consensus opinion of two examiners. Similar to proficiency test procedures, the final decision agreed upon by the primary and second examiners is reviewed for satisfaction. Documentation of consultations is maintained in the case record.

During this study, 11 firearms examiners were actively participating in casework at HFSC. Six (55%) of the examiners were trained by the Houston Police Department Crime Laboratory prior to the formal inception of the Houston Forensic Science Center in April 2014. The remaining five examiners were primarily trained by other agencies or programs. During this time frame, six examiners were certified by AFTE. In addition, all examiners were required to be licensed by the Texas Forensic Science Commission as of January 1, 2019. Examiner experience ranged from 5.5 to 23 years, with a median of 11.5 years. Table 2 shows examiner experience, training, and certification details.

**TABLE 2:** Examiner experience, training, and certification

Examiner	Experience (Years)	Original Training Lab HPD	AFTE Certification
1	12	Yes	Yes
2	23	Yes	Yes
3	7	No	No
4	5.5	Yes	No
5	22	Yes	Yes
6	12.5	Yes	No
7	7	No	No
8	17.5	No	Yes
9	8	No	No
10	7	Yes	Yes
11	11	No	Yes
	Median = 11.5		

### 2.3 Statistical analysis

Statistical analysis was performed using JMP version 13.2.1. Categorical data for the satisfactory rating were converted to continuous data on a numeric scale, so the means could be analyzed with a one‐way analysis of variance (ANOVA). A one‐way ANOVA test was performed to determine whether factors such as complexity of the case or evidence type had a statistically significant difference in the means of these reported results. The data were considered significant for *p* < 0.05.

The data set yielded two results overall. The reported results either matched the ground truth or resulted in an inconclusive decision. All comparisons were deemed satisfactory; however, the results were further analyzed to gain more insight into the inconclusive decisions. To do this, a numeric value of 100 was assigned to the satisfactory rating when the ground truth was the reported result and a numeric value of 0 was assigned when the reported result was inconclusive. Unsatisfactory results would have been assigned a numerical value as well, but because no unsatisfactory results were observed, additional values were not requisite. Once the data were in a continuous format, the numeric results of the conclusions in the data set could be used to obtain the average and allowed for the application of statistical analysis. With the numeric scale used, one can expect that a lower mean indicates that there were more inconclusive results in the data set.

## 3 RESULTS

The results of 51 blind QC cases were reported between December 21, 2015, and June 22, 2021. Most cases contained a handful of evidence items, while some contained as little as two items or as many as 41 items (median = 9). Five hundred and fourteen evidence items were submitted; however, not all items, like unfired evidence or magazines, were examined as a part of routine casework. Four hundred and sixty items, including test fires created as part of routine casework, were examined. A total of 570 sufficiency determinations and comparison conclusions were made. Because the data were examined at the comparison level, an item of evidence can appear in the data set in multiple comparisons and be represented by multiple comparison conclusions. For example, Item 1 may have been compared to Item 2 and Item 3 with comparison conclusions of elimination and identification, respectively. Thus, a case may contain more comparisons than reported conclusions.

The mock evidence items were created with ground truth being 67.7% (*n* = 386) identification, 25.0% (*n* = 143) elimination, 2.0% (*n* = 11) insufficient, and 3.2% (*n* = 18) unsuitable. Due to the small sample size, insufficient and unsuitable conclusions will be grouped together for the purposes of this study. Ground truth was unable to be determined for 2.1% (*n* = 12) of the conclusions that were reported as inconclusive. The items were bullet items that were either not marked with a UV pen prior to submission or the pen markings rubbed off during examination. The firearms section manager conducted a post‐analysis comparison on the items but was unable to reach a conclusion of identification or elimination, supporting the examiners' conclusions of inconclusive. The 12 unknown ground truth bullet item comparisons were excluded from analysis. Table 3 shows the data totals used in this study.

**TABLE 3:** Data totals used for this study

Evidence Type	Number of Comparisons	Ground Truth ID	Ground Truth Elim	Ground Truth Insuf/Un	Reported Ground Truth	Reported Inc; Ground Truth ID	Reported Inc; Ground Truth Elim
Bullet Items	272	192	72	8	104	109	59
Cartridge Cases	265	194	71	0	208	10	47
Fragments	18	0	0	18	18	0	0
Shot Carrier/Pellet	3	0	0	3	3	0	0
Total	558	386	143	29	333	119	106

*Note*. Twelve (12) bullet item comparisons were excluded from the results because the ground truth was unknown. Bullet items include bullets and bullet jacket fragments suitable for comparison. Fragments include bullet cores and nondescript metal pieces where the ground truth was unsuitable or insufficient.

Abbreviations: Elim, elimination; ID, identification; Inc, inconclusive; Insuf, insufficient; Un, unsuitable.

Satisfactory results were obtained for all items evaluated, or, by the “hard error” definition [25], no hard errors were observed; that is, no identifications were declared for true nonmatching pairs, and no eliminations were declared for true matching pairs. The ground truth was compared to the examination result, and the ground truth was obtained in 59.7% (*n* = 333) of the comparisons. In 40.3% (*n* = 225) of the comparisons, an inconclusive conclusion was made when the ground truth was either elimination or identification. A ground truth of elimination and comparison result of inconclusive was observed more frequently at 74% (*n* = 106), while the ground truth of identification and comparison result of inconclusive was observed at a rate of 31% (*n* = 119). All items submitted as ground truth insufficient or unsuitable were satisfactorily determined as such. Furthermore, no ground truth submissions of identification or elimination were determined to be unsuitable or insufficient. Inconclusive decisions were only reported for items with a known ground truth of identification or elimination; however, due to the quality of the evidence submitted, inconclusive results were not unexpected. Table 4 shows the distribution of blind QC cases and casework conclusions by examiner. Shot pellets (*n* = 1) and shot carriers (*n* = 2) were excluded from the data set because this evidence was not compared.

**TABLE 4:** Examiner blind QC case distribution and casework conclusions

Examiner	As Primary Examiner			As Second Examiner			Type of Evidence			Ground Truth		Conclusion	
	Cases	Items	Comparisons	Cases	Items	Comparisons	Cartridge Cases	Bullet Items	Fragments	ID	Elim	Ground Truth Reported	Ground Truth Reported as Inc
1	9	93	148	6	74	98	72	70	6	88	53	62	86
2	2	16	12	4	32	28	8	2	2	10	0	10	2
3	4	47	50	7	62	65	22	27	1	38	8	35	15
4	2	7	14	2	16	13	0	14	0	6	8	5	9
5	2	46	62	15	164	162	5	57	0	58	4	12	50
6	3	17	18	1	20	19	9	9	0	18	0	13	5
7	8	107	105	4	30	27	56	43	6	64	34	72	33
8	11	98	81	5	51	91	51	29	1	55	22	61	18
9	1	6	6	2	12	20	6	0	0	3	3	6	0
10	8	67	65	5	53	47	30	30	2	39	9	48	7
11	1	10	9	0	0	0	6	3	0	7	2	9	0
Total	51	514	570	51	514	570	265	284	18	386	143	333	225

*Note*. Ground truth was unknown for twelve (12)] bullet item comparisons. Shot pellets (*n* = 1) and shot carriers (*n* = 2) were excluded from the data set. Bullet items include bullets and bullet jacket fragments suitable for comparison. Fragments include bullet cores and nondescript metal pieces where the ground truth was unsuitable or insufficient.

Abbreviations: Elim, elimination; ID, identification; Inc, inconclusive.

The data were examined at the comparison level, so the number of inconclusive conclusions may appear to be inflated when compared to casework rates. One item of evidence may have been determined to be inconclusive to multiple items of evidence, consequently appearing in the data set more than once. The inconclusive conclusions rendered were evaluated for trends. Data factors such as evidence type and complexity of the comparison were evaluated to determine whether these factors contributed to a higher rate of inconclusive results.

The data showed that evidence type significantly contributed to inconclusive conclusions. Specifically, bullet items (61.8%; *n* = 168) were the main contributor and then cartridge cases (21.5%; *n* = 57). When comparing the means between the bullet items (38.235) and cartridge cases (78.491), bullet items had a lower mean, which indicates more inconclusive conclusions since these conclusions were assigned a 0 in the data set. The difference between these means was statistically significant. Table 5 shows the outcomes for comparisons based on evidence type grouping, again excluding shot pellets (*n* = 1) and shot carriers (*n* = 2).

**TABLE 5:** Outcomes for comparisons based on evidence type grouping

Evidence Type	Number	Mean	SE	Lower 95%	Upper 95%
Bullet Items	272	38.235	2.694	32.943	43.53
Cartridge Cases	265	78.491	2.729	73.129	83.85
Fragments	18	100.00	10.473	79.429	120.57
*F* Statistic	*F* Ratio = 62.8332		Prob > *F* = < 0.0001*		

*Note*. Twelve (12) bullet item comparisons were excluded from the results because the ground truth was unknown. Shot pellets (*n* = 1) and shot carriers (*n* = 2) were also excluded from the data set. Bullet items include bullets and bullet jacket fragments suitable for comparison. Fragments include bullet cores and nondescript metal pieces where the ground truth was unsuitable or insufficient. *Indicates significance at less than 0.01.

Furthermore, the comparisons in which ground truth was elimination but reported as inconclusive break down as 59 bullets and 47 cartridge cases. Conversely, the comparisons in which ground truth was identification but reported as inconclusive break down as 109 bullets and 10 cartridge cases. When evaluating the complexity of the comparison, 51% of all comparisons were created with two different firearms of the same class making the comparisons more challenging with identical class characteristics. For bullets, all the elimination conclusions were determined on class characteristics. When comparing the means between the evidence created using the same firearm or not, there was not a significant difference, which indicates the complexity of the case did not significantly contribute to the inconclusive conclusions. Table 6 shows the outcomes for comparisons created from two firearms of the same class.

**TABLE 6:** Outcomes for comparisons created from two firearms of the same class

Two Firearms of the Same Class	Number	Mean	SE	Lower 95%	Upper 95%
No	280	59.2857	2.9367	53.517	65.054
Yes	278	60.0719	2.9473	54.283	65.861
*F* Statistic	*F* Ratio = 0.0357	Prob > *F* = 0.8502			

*Note*. No significance noted.

Initially, one examiner pairing did appear to have a significant difference in inconclusive rate. However, after further evaluation this is attributed to the number of cases and number of bullet items assigned to the examiner (Table 4). The distribution of cases to the primary and second examiners is not normal; therefore, an examiner assigned cases with more bullet items would have more inconclusive conclusions. For example, Primary Examiner 1 appears to have the majority of inconclusive decisions; however, this examiner completed 148 comparisons, 70 of which were bullet items.

In nearly all blind QC cases, the primary and second examiners agreed on the examination conclusions. Since the implementation of the firearms section's consultation and conflict resolution policy [22] in 2018, two consultations were documented in the blind QC program within the timeframe of this study. Only one consultation was a result of a difference in comparison conclusions between the primary and second examiners. Neither consultation rose to the level of a conflict resolution, and the primary and second examiners involved in each case were able to reach consensus agreement.

In the first case, the primary examiner sought consultation regarding three bullets prior to verification by the second examiner. The primary examiner was unsure if the markings on the bullets were individual in nature. Together, the examiners decided the markings were individual and could be used for identification. The three bullets fired from the same firearm were the only items submitted for this blind QC, and the ground truth was in fact identification.

In the second case, the primary examiner made an inconclusive decision between two bullets. During verification, the second examiner made an identification conclusion. The primary and second examiners microscopically reviewed the items and discussed the observed markings. Due to the distorted condition of the items and the overall quality of the markings, the examiners together decided on an inconclusive decision. The firearms section manager reviewed this case upon completion and confirmed that the items were stretched and distorted, rendering the analysts' inconclusive conclusion appropriate. This case also involved a consultation on three cartridge cases; the second examiner opined that more areas of agreement were needed to make an identification on the cartridge cases. The primary examiner agreed, and the additional areas of agreement were documented in the case record. A total of 12 items (eight cartridge cases, two bullet items, and two fragments) were submitted for this blind QC, all fired from the same firearm. The ground truth for all items was identification, with the exception of the two fragments, which were correctly concluded to be insufficient for examination.

## 4 DISCUSSION

The results presented here represent preliminary outcomes from a blind testing program in firearms examination over c. five and a half years. HFSC's blind QC program inserts mock firearms cases into the sectional workflow to mimic real casework, and the outcomes offer a glimpse into the complete process of firearms examination, from evidence submission to reporting of results. Notably, the results reflect outcomes when examiners were truly blind (i.e., unaware that they were completing a test and not genuine casework).

Firearms examiners determined the correct ground truth result in over half (~60%) of comparisons. Inconclusive determinations were reached for 40% of comparisons; ground truth eliminations resulted in a higher rate of inconclusive responses (74%) than ground truth identifications (31%). This could be because identifications are generally considered easier to make based on individual characteristics alone. When an examiner arrives at an elimination conclusion, the examiner has determined that the examined items being fired in the same firearm would be, in their opinion, a practical impossibility. When class characteristics differ, elimination is the appropriate choice, by definition. However, when observable class characteristics do agree, the examiner must consider how variables such as differences in ammunition and condition of the bearing surfaces of the firearm (e.g., barrel and breechface) impact the individual characteristics imparted on fired bullets and casings.

Lack of sufficient agreement of individual characteristics could be due to the previously mentioned factors (and others, such as incomplete/damaged bullets) or to the items being fired in different firearms. Given that the burden of proof for an elimination conclusion is greater than that of identification, a more prudent conclusion could be inconclusive when the examiner cannot rule out factors that contribute to insufficient agreement of individual characteristics. HFSC firearms examiners can make conclusions of elimination on individual characteristics without having a firearm submitted; however, when a firearm is submitted, the examiners can use the firearm to create additional test fires and examine the bearing surfaces of the firearm. An elimination decision may be easier to conclude when a firearm is submitted.

Inconclusive conclusions did not appear to be related to primary and second examiner pairings, examiner experience level, or the examiners' primary training locations. Most examiners are long‐stay examiners and have been working under the same procedures for years; thus, these results are not surprising. A little over half (51%) of the cases were created using two different firearms of the same class with the intent of making comparisons more challenging; however, this variable was shown to be an insignificant source of inconclusive results.

Breaking down the inconclusive conclusions by evidence type showed that comparisons of bullet items resulted in inconclusive conclusions more often than cartridge cases (~62% and ~22%, respectively). When conducting microscopic comparisons on bullets, there are fewer areas of interest at which to look (e.g., land impressions) because only one part of the firearm contacts the bullet (barrel). Cartridge cases are contacted by more parts of the firearm, therefore providing more surfaces (i.e., breechface, firing pin, chamber and, to a certain extent, the ejector/extractor) and more information with which to make comparison conclusions. The brand of ammunition used can contribute significantly to differences in individual marks. Comparisons can be easier if the same brand of ammunition was used. Since the brand is typically discernable on cartridge cases, examiners can use the same brand of ammunition to create test fires, making comparisons easier. However, bullet comparisons can be more challenging because the brand of bullet is typically not discernable. Similar to Smith et al. [25], these results indicate firearms examiners routinely reach a correct determination of ground truth identification for cartridge cases and bullets (more sensitivity) but may have more difficulty discriminating elimination in bullets compared with cartridge cases (less specificity).

As the results show, not all comparison conclusions are simply either identification or elimination. Neither HFSC nor AFTE considers inconclusive decisions to be errors or “hard errors” as defined by Smith et al. [25]. Rather, an inconclusive decision can be viewed as an analog of analytical variability and conceptually a framework of sensitivity and specificity of the analysis. An examiner must determine “sufficient agreement” to make an identification and “sufficient disagreement” to determine an elimination, per the AFTE range of conclusions [20] and the firearms section range of conclusions document [21]. If some agreement (or some disagreement) exists, but the examiner cannot attribute that agreement (or disagreement) to the items being fired in the same gun (or different guns), then the examiner will conclude inconclusive. A multitude of factors may impact the physical evidence making the decision to identify or eliminate more difficult. Time, damage to the firearm, brand of ammunition, and the ability to switch firearm parts can all effect examination results. The firearm may not be submitted in the same condition as when used in a crime. Furthermore, groove impressions on bullets are prone to subclass characteristics. If subclass cannot be ruled out, the examiner must make an inconclusive determination.

Equating inconclusive determinations to not being able to exclude is inappropriate. The results suggest that examiners more easily identify cartridge cases than bullets, but an inconclusive result for comparison of either evidence type may be a ground truth of identification or elimination. Alternatively, an inconclusive decision means that not enough information is available to allow the examiner to conclude either identification or elimination. The distinction of the simple false positive or false negative as “hard errors,” as is done in many studies, does not account for all the possibilities in which the known ground truth might not be concluded. An inconclusive decision when a ground truth result is intended must also be examined as this could indicate a form of error in the analytical process. However, inconclusive conclusions must remain a viable result because the limits of currently used technology and test methods are not specific or sensitive enough to render a ground truth result in every situation. The results suggest that inconclusive results in firearms examination could be considered as analogous to a detection limit in a quantitative toxicology analysis.

If a primary examiner makes an inconclusive decision, the reason for the conclusion must be explained in the case record. A second examiner will verify and agree or disagree with the decision. If the second examiner disagrees, the firearms section's consultation and conflict resolution policy [22] will be followed, and an agreement will be reached. Although not ideal, the current method of firearms examination makes inconclusive decisions necessary. If a firearms examiner was required to reach only identification or elimination conclusions, examination may result in errors.

While blind QC cases are intended to mimic actual casework in packaging and offense type, the program does provide a unique opportunity to submit challenging cases that test the examiners' abilities. Philosophically, HFSC has chosen to specifically target challenging case scenarios in an effort to better define the limitations of analysis. Submitting “easy” comparisons in which the examiners are more likely to conclude elimination or identification instead of inconclusive would not make blind QC cases distinct from open proficiency tests. Focusing on challenging cases may result in blind QC data that does not accurately represent inconclusive rates in real casework. Rather, the intent is to have blinds be more sensitive than casework to sources of variance.

### 4.1 Limitations

In the initial stages of the program, record keeping was inconsistent until the submission process became more comfortable. Which firearm was used to create the fired evidence was not consistently recorded, thus leaving gaps in understanding if the fired evidence was created using a firearm that produced robust or indistinct marks. In addition, while most items have a ground truth of identification or elimination, the manager preparing the evidence items has an idea about whether the examiner will conclude the ground truth or make an inconclusive determination. However, the ground truths for potential inconclusive determinations were not consistently documented, which made data analysis challenging. Moving forward, potentially inconclusive items should be documented or marked with a UV pen to better evaluate the data in future.

Limitations also exist in resource availability. The most easily accessible source of firearms that can be used to create fired evidence is HFSC's reference collection; however, the collection does not contain all the firearms that are commonly seen in real casework. Firearms that are slated by HPD for destruction and HFSC personnel‐owned firearms can also be used to create fired evidence, but the firearms available through either source is limited in diversity and quality. One advantage to having access to HPD‐destruction firearms is being able to submit in blind QC cases firearms that examiners will not recognize, which would be the case if a reference collection firearm was submitted.

### 4.2 Benefits

The firearms section at HFSC benefits from the blind QC program in several ways. Because the program is an extension of the open proficiency testing program, the section can more fully gauge their processes in the areas that the proficiency tests do not cover while still providing a ground truth. For example, proficiency tests typically consist of only bullets or only cartridge cases for microscopic comparisons, but blind QC cases can include both bullets and cartridge cases, as well as fragments and firearms. Blind QC cases also have the potential to test caliber determinations and trigger pull measurements. This allows the laboratory to be tested in areas on the scope of accreditation that proficiency tests cannot meet. Compared with proficiency tests, more challenging blind QC cases can be created to test the examiners' thresholds for determinations. Since blind QC cases mimic actual casework and are conducted blind, the cases allow for a more accurate and effective measure for how examiners and processes and procedures are operating. Measuring the entire workflow could provide the section with a way to potentially discover bottlenecks or areas for improvement in their processes and procedures, which is more difficult to do with proficiency tests and real casework. Furthermore, regular participation in the program gives the examiners the opportunity to bolster their credibility in court when testifying on real cases.

### 4.3 Future directions

In November 2015, the firearms section implemented a blind verification procedure for select cases. In a typical case, the primary examiner's conclusions are visible to the second examiner. In a blind verification, the primary examiner's conclusions are masked from the second examiner, allowing the second examiner to conduct an independent examination with minimized bias from the primary examiner's conclusions. Blind verifications are selected by section management at a rate of one case per month and can be performed on real casework or blind QC cases. In future, HFSC would like to examine trends in blind verification cases as well the rates of inconclusive conclusions and consultations and conflicts in real casework.

The firearms examination community will benefit from a more focused and narrowed experimental design. For example, formal research on the ability of firearms to leave distinct marks on bullets and casings could be used in court when testifying to inconclusive results. Deliberately creating and submitting evidence from firearms that do not mark well could be an advantageous and more specific route for the blind QC program to take next. Blind QC cases could be constructed well ahead of time and submitted with intention, depending on the research question being asked.

Blind QC cases can be utilized for training new examiners or determining the efficacy of new technology. HFSC is in the early stages of exploring the use of a 3D imaging instrument in firearms examination. 3D imaging may make visible previously imperceptible details, which can be usable data points in comparisons. Using blind QC cases for this study will help determine whether 3D imaging technology will impact the firearms comparison practice. If firearms procedures or methods change, the blind QC program will inevitably have to change as well; thus, the program and possible research evolve naturally with the discipline.

The Houston Forensic Science Center would also like to collaborate with other laboratories to expand the availability of firearms and ammunition to submit as blind QC cases. Not only would collaborating with other laboratories provide a bigger selection of evidence, but multiple laboratories could use test fires created from the same firearms providing opportunities for between‐laboratory comparisons of blind testing results. Another future direction is to collaborate with researchers to study rates of inconclusive decisions. Further studies could help address criticisms aimed at inconclusive decisions as well as provide a current standard in the field to determine whether new technologies (e.g., 3D imaging) assist with inconclusive decisions. Such studies could help better define and improve sensitivity and specificity of firearms examination.
